# Tournaments between markers as a strategy to enhance genomic predictions

**DOI:** 10.1371/journal.pone.0217283

**Published:** 2019-06-24

**Authors:** Diógenes Ferreira Filho, Júlio Sílvio de Sousa Bueno Filho, Luciana Correia de Almeida Regitano, Maurício Mello de Alencar, Rosiana Rodrigues Alves, Sarah Laguna Conceição Meirelles

**Affiliations:** 1 Departamento de Ciências Econômicas e Exatas, Universidade Federal Rural do Rio de Janeiro, Três Rios, Rio de Janeiro, Brazil; 2 Departamento de Estatística, Universidade Federal de Lavras, Lavras, Minas Gerais, Brazil; 3 Embrapa Pecuária Sudeste, São Carlos, São Paulo, Brazil; 4 Embrapa Pesca e Aquicultura, Palmas, Tocantins, Brazil; 5 Departamento de Zootecnia, Universidade Federal de Lavras, Lavras, Minas Gerais, Brazil; Jaypee University of Information Technology, INDIA

## Abstract

Analysis of a large number of markers is crucial in both genome-wide association studies (GWAS) and genome-wide selection (GWS). However there are two methodological issues that restrict statistical analysis: high dimensionality (*p*≫*n*) and multicollinearity. Although there are methodologies that can be used to fit models for data with high dimensionality (eg, the Bayesian Lasso), a big problem that can occurs in this cases is that the predictive ability of the model should perform well for the individuals used to fit the model, but should not perform well for other individuals, restricting the applicability of the model. This problem can be circumvent by applying some selection methodology to reduce the number of markers (but keeping the markers associated with the phenotypic trait) before adjusting a model to predict GBVs. We revisit a tournament-based strategy between marker samples, where each sample has good statistical properties for estimation: *n*>*p* and low collinearity. Such tournaments are elaborated using multiple linear regression to eliminate markers. This method is adapted from previous works found in the literature. We used simulated data as well as real data derived from a study with SNPs in beef cattle. Tournament strategies not only circumvent the *p*≫*n* issue, but also minimize spurious associations. For real data, when we selected a few more than 20 markers, we obtained correlations greater than 0.70 between predicted Genomic Breeding Values (GBVs) and phenotypes in validation groups of a cross-validation scheme; and when we selected a larger number of markers (more than 100), the correlations exceeded 0.90, showing the efficiency in identifying relevant SNPs (or segregations) for both GWAS and GWS. In the simulation study, we obtained similar results.

## Introduction

Technological advances in the genotyping process have allowed the development of new molecular marker classes, among which single nucleotide polymorphism (SNP) stands out. One of the main advantages of this type of marker is the possibility of simultaneous genotyping of thousands of SNPs, widely distributed throughout the genome, such that some of these markers will probably be in linkage disequilibrium with quantitative trait loci (QTLs).

Multiple linear regression models can be used to describe the relationship between phenotypes and markers. The marker effects are estimated from the model and, based on these estimates, hypothesis tests and predictions can be performed [1]. Hypothesis tests are used to identify SNPs with statistical significance (SNPs in linkage disequilibrium with QTLs). From marker effect estimates (adjusted model), one can predict genomic breeding values (GBVs) of individuals for genomic selection.

Genome-wide selection (GWS), proposed by [2], consists on estimate effects of thousands of markers to predict GBVs and, based on these values, identify genetically superior individuals. In GWS, effects of all markers are simultaneously estimated, without previously identifying significant markers [3]. However, the large number of markers leads to problems of collinearity (different markers with the same genotypic profile) and dimensionality (number of markers much larger than the number of genotyped individuals) [4]. In this situation, the ordinary least squares method becomes inapplicable, and other statistical methods are needed. Among the statistical methods used in GWS we can mention: RR-BLUP, G-BLUP, Elastic Net, Partial Least Squares, Bayes A, Bayes B and Bayesian Lasso. Among them, one of the most prominent is Bayesian Lasso which is a bayesian formulation of the Lasso method. Briefly, the Lasso (Least Absolute Shrinkage and Selection Operator) is a penalized regression method, originally proposed by [5]. A Bayesian formulation (Bayesian Lasso) was made by [6]. Bayesian Lasso has been used in several association studies [7, 8] and in genomic selection [9–11].

One of the great advantages of marker assisted selection is the possibility of reducing the time between generations through the early evaluation of individuals, i.e., using a model that was fitted using adult individuals, and inserting in the model information from molecular markers of young individuals (who dont were used to fit the model), we can predict the GBVs of these young individuals in adulthood. However, [12] highlights the fact that using thousands of markers to fit a model can lead to overfitting, i.e., errors in the data can be explained by marker effects. In this case, the model’s predictive ability should perform well on a training set (individuals used to fit the model), but should not perform well on a testing set (individuals that were not used to fit the model), restricting the applicability of the model.

To assess the model’s predictive ability, one can use the cross-validation technique. [13] proposed fitting a model using all markers (full model) and getting estimates of each marker. Using different numbers of markers (100, 250, 500, 1,000,…) that obtained the highest estimates (in absolute value) in the full model, new models are fitted, which are then used for prediction of GBVs. Using a cross-validation scheme one can adjust a model (estimate the markers effects) from a training set and use this model to predict GBVs of individuals of a testing set. Then, the correlations between the predicted GBVs and observed phenotypes in the testing set can be obtained. The individuals in the testing set were not used to fit the model, so the correlation between predicted GBVs and phenotypes in the testing set is due to genetic factors. Thus, this correlations are used to estimate the approximate number of markers that will provide better predictive ability. In this case, the choice of marker subsets is based on classification of the markers by the absolute values of their estimates in the full model. However, this approach requires obtaining a full model, and this requires a great deal of time and computational effort, without assurance of solving the problem of multicollinearity, which is very common in this kind of study.

An alternative method that can be used to help select markers and has better computational performance is tournament screening proposed by [14]. This method basically consists of forming random groups of markers and, as in a tournament, selecting the top markers of each group at each stage. The markers selected from the groups go on to the next stage of the tournament, where they are again reunited in a single group and later separated into new random groups from which the top markers will once more be selected. The underlying hypothesis is that marker selection by tournaments can be a way to mitigate the problem of multicollinearity because the formation of groups for marker selection decreases the probability of markers with the same segregation in each analysis. Another advantage of this method is that it allows a more efficient computational approach because analyses in each group are much faster than analysis using all markers. In addition, large-scale data processing can often achieve better performance using parallel programming. In the tournament method, the analyses in each group are independent; thus, they can be carried out simultaneously using parallel programming.

We proposed a method to help marker selection by using tournaments of multiple linear regression analyses. The central idea is to form random groups of markers and subject each group to traditional multiple linear regression analysis. The marker that obtains the highest *p*-value in each group (worst marker of each group) is eliminated, and the markers remaining from all groups (those that were not eliminated) are clustered into a single group and are later used to form new random groups in the next stage of the tournament. The process repeats until the number of markers is reduced to a desirable level. After the number of markers has been reduced to a desirable level, we can apply another method of marker selection to make a final selection. For that purpose, we fitted a model using Bayesian Lasso and selected the statistically significant markers by the credibility intervals of the markers.

Considering this method and taking into account the large number of SNPs in each chromosome, the hypothesis arises that forming marker samples conditioned to the chromosome structure can further reduce multicollinearity effects over the regression analysis and provide more security in the marker selection process.

The main objective of this study was to evaluate if the use of tournaments with multiple linear regression can improve marker selection. Note that this method dont provide a final selection of markers, this method only reduce the full set of markers (keeping the markers associated with phenotype) improving the further aplication of another selection method. We also verified if the way the groups are formed (random or chromosome-conditioned groups) in tournaments influences marker selection and GBV prediction. Both tournaments (with random and chromosome-conditioned groups) were compared to Bayesian Lasso, which was used as a reference. Simulation studies and real data analysis were performed.

The choice of Bayesian Lasso as the reference method was made because it is a very used method in GWAS and GWS and with this method it is possible to choose the quantity of markers to be selected (using as criterion the size of the markers effects estimates) and then to compare these selected markers with the same quantity of markers selected by tournaments. Another reason to use Bayesian Lasso as a reference method is because in real data analysis we choose Bayesian Lasso as the final procedure to select markers using credibility intervals, regardless of the methodology used to reduce the full set of markers (Tournament or Bayesian Lasso).

### Tournament screening

The following steps make up the tournament method:

- Step 1: The set of all markers, *S*_1_, is partitioned into smaller *k* groups: *S*_11_ ∪ *S*_12_∪ … ∪ *S*_1*K*_, each group of approximately equal size *p*_*g*_, *p*_*g*_<*p*, where *p* is the total number of markers. Using the markers of each group, a classical linear regression model is fitted in which each marker is considered an explanatory variable *x* of the model, as presented in Eq (1):
  $$\begin{array}{c} y_{i}=\mu +\sum_{j=1}^{p_{g}}x_{ij}\beta_{j}+\varepsilon_{i} \end{array}$$ (1)
  where *y*_*i*_ (*i* = 1, 2, …, *n*) is the phenotype of the individual *i*; *μ* is the overall mean; *x*_*ij*_ is the genotype of the SNP *j* (*j* = 1, 2, …, *p*_*g*_) of the individual *i*, encoded as 0, 1 or 2, based on the number of copies of one of the SNP alleles; *β*_*j*_ is the main effect of the SNP *j* and *ε*_*i*_ i is the random error, $\varepsilon_{i}∼N\left(0,\sigma_{\varepsilon }^{2}\right)$.
  After estimating the marker effects of the group *S*_1*k*_ (*k* = 1, 2, …, *K*), the marker whose estimate had the highest p-value is eliminated or, in the case of perfect collinearity, one of the markers whose effect has not been estimated is eliminated randomly. Then, the markers selected (those that were not deleted) of all groups are clustered into a single larger group: *S*_2_.
- Step 2: Repeat the procedure of Step 1, substituting *S*_1_ for *S*_2_.
- Further steps: Repeat the above steps until the total number of markers is reduced to the desired level.

During the tournament stages, the sequence of elimination of the markers can be stored, generating a marker score, so the most important markers are those that remained at the end of the tournament, followed by the markers eliminated in an order that starts from the markers eliminated last to those that were eliminated first in the tournament. Thus, if the final number of markers selected by the tournament is small and a larger number of markers is desired, it is not necessary to perform another tournament; an additional number of these markers already selected can simply be added according to the classification generated by the order of elimination.

The time for executing the tournament method can be substantially reduced using parallel programming, since the analyses in each group are performed independently.

## Materials and methods

### Datasets

#### Real genotype data and quality control

The SNP genotype data used in this study came from a real bovine study conducted by Embrapa Pecuária Sudeste. A group of 400 animals of the Canchim breed was genotyped with BovineHD BeadChip chips (Illumina Inc., San Diego, CA, USA). The original data set contained genotypes of nearly 800,000 SNPs of 400 animals. A data quality control step was performed, which included the elimination of samples (animals) and SNPs. Sample quality was controlled by eliminating samples with a call rate of less than 98%, requiring heterozygosity in relation to the mean of the samples greater than three standard deviations, and identifying outliers by plotting multidimensional scaling (MDS). Quality control of markers included elimination of those with missing genotypes, those with allele frequency of less than 3%, fixed genotypes in the population (100% occurrence of a single genotype),and redundant markers (markers with identical genotypic profiles). Redundant markers were verified only for adjacent markers. After quality control, 384 animals and 530,566 SNPs remained in the study.

#### Real phenotypes

The real phenotypic character studied was rib eye area (REA). To perform the analyses, the phenotypic data were corrected to the contemporary group effects. To form the contemporary group, 987 animals were used, including 384 of the 400 genotyped animals; the contemporary groups were formed by the following variables: year of birth, farm, genetic group, and sex. The corrected phenotypes are the residuals of a fixed effect model of the REA variable as a function of the above-mentioned variables.

#### Simulated SNP effects

**Fig 1:**
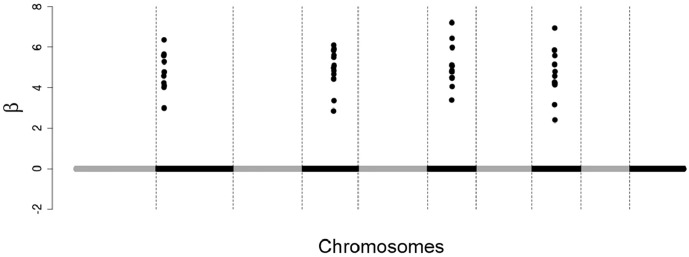
Simulated effects of SNPs. 48 SNPs with non-zero effects, distributed among 4 chromosomes, close to each other on the chromosome.

**Fig 2:**
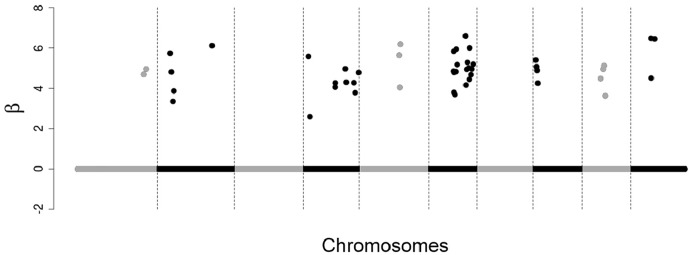
Simulated effects of SNPs. 48 SNPs with non-zero effects, distributed among eight chromosomes, a little more dispersed from each other on the chromosome.

**Fig 3:**
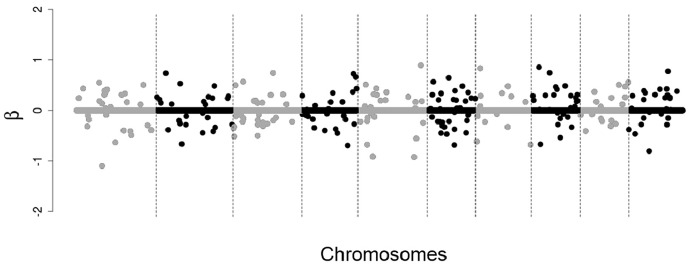
Simulated effects of SNPs. 250 SNPs with non-zero effects, distributed throughout the chromosomes, dispersed from each other on the chromosome.

To verify marker selection ability from the tournament method, marker effects were simulated. To simulate marker effects, the real map of 384 animals of the Canchim breed was used. The genotype data set used in the simulation study consisted of 11,812 SNPs chosen from chromosomes 20 to 29. The genotype data set was limited in the simulation study to reduce time of analysis and to enable several replications of the analyses. Vectors of SNP additive genetic effects (*β*) containing a certain number of SNPs with non-zero effects and the remainder with zero effects were simulated. These vectors were simulated considering three different situations:

1. 48 SNPs with non-zero effects, distributed among four chromosomes, close to each other on the chromosome (Fig 1);
2. 48 SNPs with non-zero effects, distributed among eight chromosomes, a little more dispersed from each other on the chromosome (Fig 2);
3. 250 SNPs with non-zero effects, distributed throughout the chromosomes, dispersed from each other on the chromosome (Fig 3).

In situations 1 and 2 (Figs 1 and 2) the objective was to simulate large QTLs for that all the methodologies evaluated could easily detect the most of them, making it easier to compare the methodologies with respect to the markers selection capacity, so we simulated only positive and close effects in these situations. In situation 3 (Fig 3) we simulate a more realistic case, that is, many small individual effects (positive and negative) dispersed by the chromosomes.

Note that the number of markers used in the simulation study is much smaller than the number of markers of the real data set. However since the number of simulated markers with non-zero effects (48 to 250 markers) is very small compared to almost 12,000 markers used in the simulation study, it is expected that this reduction in the set of markers for the simulation study dont be a problem to the comparisons between the methodologies since for one methodology to be superior to another it must to be able to select a bigger number of markers with non-zero effects, regardless of the other markers with null effects.

#### Simulated GBVs and phenotypes

Based on the simulated effects vector (*β*) of SNPs and the real incidence matrix of the SNP genotypes (*X*), the GBVs of the animals were obtained: *GBV* = *Xβ*. From these GBVs, phenotype vectors of the animals were simulated according to Eq (2):

$$\begin{array}{c} y=1\mu +X\beta +\varepsilon \end{array}$$ (2)

where *μ* is the general mean common to all observations (we consider *μ* = 100), and *ε* is the residue vector normally distributed with zero mean and variance $\sigma_{\varepsilon }^{2}$.

Phenotype vectors were simulated considering three different heritabilities: 0.25, 0.5 and 1.0. The residue vectors (*ε*) were simulated according to a normal distribution with zero mean and variance $\sigma_{\varepsilon }^{2}$, compatible with the desired heritability. To obtain $\sigma_{\varepsilon }^{2}$, we isolated it in Eq (3):

$$\begin{array}{c} h^{2}=\frac{\sigma_{GBV}^{2}}{\sigma_{GBV}^{2}+\sigma_{\varepsilon }^{2}} \end{array}$$ (3)

where *h*^2^ is the heritability, and $\sigma_{GBV}^{2}$ is the additive genetic variance (variance of *Xβ*). Thus, for *h*^2^ = 0.25, one understands $\sigma_{\varepsilon }^{2}=3\sigma_{GBV}^{2}$, and for *h*^2^ = 0.50, one understands $\sigma_{\varepsilon }^{2}=\sigma_{GBV}^{2}$. To simulate *y* with heritability *h*^2^ = 1.0, no residue vector (*ε*) was used in Eq (2). Heritability *h*^2^ = 1.0 was used to evaluate the limits of the method in the absence of experimental error.

### Marker selection

A marker was considered correctly selected if it was an SNP whose effect was simulated with non-zero effect or if it was close to a non-zero effect SNP.

Graphical analysis of the selected markers was made to subjectively determine whether or not they were close to the actual SNPs of non-zero effects. For that purpose, the marker selection stage was repeated 100 times in each of the situations considered in the simulation study, and graphs of the frequencies with which the markers were selected in the 100 repetitions were made.

A method was considered superior to the others with respect to marker selection when the frequency of detection of the non-zero effect SNPs (or near to these) is greater. Markers were selected using the tournament method and also using Bayesian Lasso. To facilitate comparison between the marker selection methods, we established the number of 100 SNPs to be selected by each method.

For implementation of parallel programming, we used the Parallel package of the R software ([15]).

#### Group formation in tournaments

Two types of group formation for the tournaments were evaluated:

1. Random groups;
2. Groups conditioned to the chromosomes.

In random groups, each group was formed by markers randomly sampled from the set of all markers. In chromosome-conditioned groups, each group was formed by markers of almost all the chromosomes sampled in proportion to the number of markers of each chromosome.

The objective was to evaluate if the way the groups are formed exerts influence on marker selection and on prediction of genetic values.

#### Group size in tournaments

The influence of group size (*p*_*g*_) on the tournament was also evaluated. Three sizes of groups were evaluated: 25, 50 and 100 markers per group.

#### Marker selection by Bayesian Lasso

We also used Bayesian Lasso to make marker selection, to compare it with the marker selection made by tournaments.

In the tournaments, we selected pre-determined quantities of markers (100 markers). Then, to compare Bayesian Lasso with the tournament method in relation to marker selection ability, we used an approach that allowed pre-determination of the number of markers to be selected by Bayesian Lasso. To do so, the effects of all markers were estimated in a complete model (with all markers) using Bayesian Lasso, and the 100 markers that obtained the highest estimates (in absolute value) in this model were selected.

### GBV prediction

At the end of the marker selection process (by tournaments or Bayesian Lasso), a set of selected markers *S* is obtained. These selected markers can be used to predict GBVs. Thus, it is necessary to estimate the effects of these markers, that is, obtain the ${\hat{\beta }}_{s}$ vector. The predicted GBV vector is obtained by Eq (4):

$$\begin{array}{c} G\hat{B}V=X_{s}{\hat{\beta }}_{s} \end{array}$$ (4)

where *X*_*s*_ is the SNP genotype matrix for the selected markers, and ${\hat{\beta }}_{s}$ is the vector of the SNP predicted effects for the selected markers.

The effects of selected markers (${\hat{\beta }}_{s}$) were estimated using Bayesian Lasso, regardless of the method used for marker selection (tournament or Bayesian Lasso). The Bayesian Lasso for estimating marker effects used a Markov chain of 4,000 iterations, burn-in of 2,000, and thin = 20.

#### Assessment of predictive ability

To evaluate the GBV predictive ability, we obtained correlations between predicted GBVs and simulated GBVs ($r_{G\hat{B}V,GBV}$) and also correlations between predicted GBVs and phenotypes ($r_{G\hat{B}V,y}$).

The models for GBV prediction were fitted using different numbers of markers. These markers were selected according to their classification in marker selection by tournaments or Bayesian Lasso. Classification of the markers was obtained by the order of elimination of the markers in the tournament; or, when Bayesian Lasso was used for marker selection, classification was obtained by the absolute value of estimates of marker effects in the complete model. Using these classifications, models were fitted for GBV prediction using Bayesian Lasso, regardless of the method used for marker selection. Models for GBV prediction were fitted using different numbers of markers: 100, 250, 500, 1,000, 2,000, 4,000, 8,000, and 11,812; and then the following correlations were calculated: $r_{G\hat{B}V,GBV}$ and $r_{G\hat{B}V,y}$.

An 8-fold cross validation was also performed to circumvent the overparametrization problem. The 384 animals were divided into 8 sets of 48 animals. In each step of cross validation, one set (testing set) was removed from the analysis and the other seven sets (training sets) were used to estimate the marker effects. From these estimated effects of markers, the animal GBVs of the testing set (which was not used to estimate the effects of markers) were predicted. Then, for the animals of the testing set, the correlations between predicted and simulated GBVs ($r_{{G\hat{B}V}_{test},GBV_{test}}$) and correlations between predicted GBVs and phenotypes ($r_{{G\hat{B}V}_{test},y_{test}}$) were calculated.

#### Analyses with real phenotypes

We analyzed the rib eye area (REA) phenotypic trait. The complete genotypic data set was used, containing genotypes of 530,566 SNPs from 384 animals. Phenotypic data were previously corrected for contemporary groups.

Markers associated with rib eye area were selected using the same marker selection methods used in the simulation study: tournaments with random groups, tournaments with chromosome-conditioned groups, and Bayesian Lasso. Tournaments were performed using only the group size (*p*_*g*_) that obtained the best performance in the simulation study.

After the marker selection stage, models for GBV prediction were fitted using the markers selected. The models for GBV prediction were fitted using Bayesian Lasso, regardless of the method used for marker selection. An 8-fold cross validation was used. Correlations between predicted GBVs and corrected phenotypes ($r_{G\hat{B}V}{}_{,y}$) were calculated for the testing set and training set of the cross validation.

Another strategy used to select the markers most associated with REA had the following steps: (1) Select 100 markers by tournament with random groups. (2) Fit a model with Bayesian Lasso. (3) Calculate 95% HPD credibility intervals to perform a final marker selection procedure. Markers whose HPDs do not include zero are selected. (4) Repeat this procedure 100 times and calculate the frequencies with which the markers are selected. (5) The markers with higher frequencies in step (4) are definitively selected.

Using the markers selected in step (5), we fitted models for GBV prediction using Bayesian Lasso, regardless of the marker selection method. An 8-fold cross validation was used. Then correlations between predicted GBVs and corrected phenotypes ($r_{\hat{GBV},y}$) were calculated for the testing set and training set of the cross validation.

#### Data availability

The phenotypic and genotypic data are available at the figshare repository and their description and accession information are listed in the S1 File. Custom R scripts used in the analysis can be found in S2 and S3 Files.

## Results

### Marker selection for simulated data

To compare the selection methods, graphs were made that allow observation of the locations of the non-zero effect markers on the chromosomes and the frequencies with which each marker was selected in 100 analysis repetitions.

**Fig 4:**
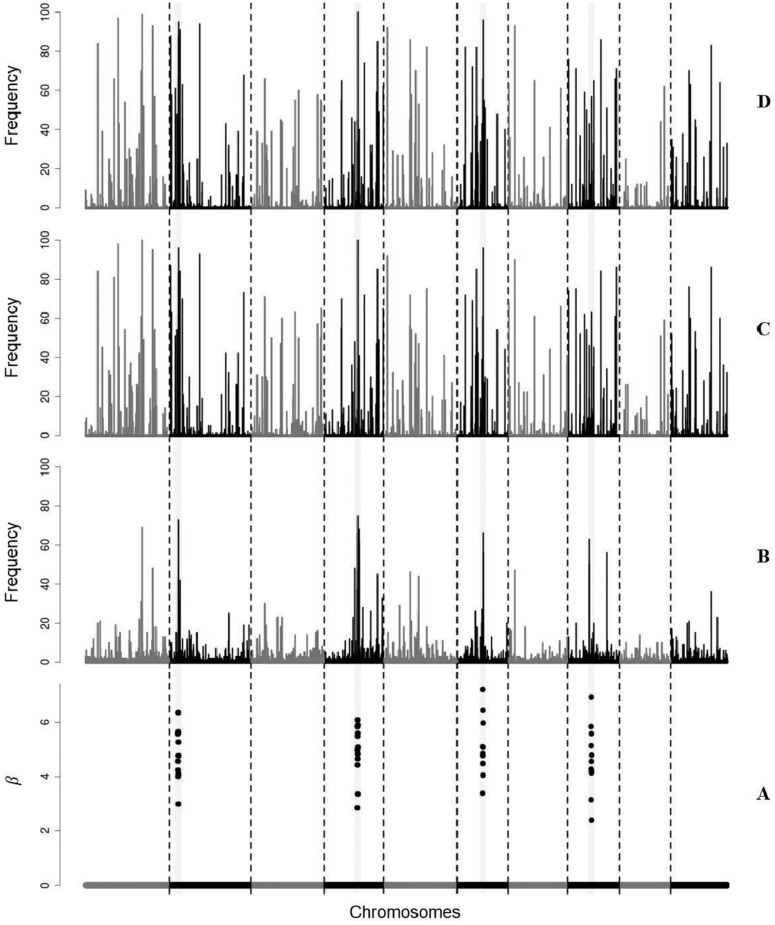
Selected SNPs in the simulation study. Simulated effects of SNPs (A) and frequencies with which the markers were selected in 100 repetitions of the selection step by the selection methods: Bayesian Lasso (B), tournaments with random groups (C), and tournaments with chromosome-conditioned groups (D).

For example, in Fig 4, we can observe the simulated effects of 11, 812 SNPs, of which 48 have non-zero effects (A), and the respective frequencies with which each SNP was selected, in 100 analyses, by each one of the selection methods: Bayesian Lasso (B), tournaments with random groups (C), and tournaments with chromosome-conditioned groups (D). The marker effects were simulated considering the scenario of 48 SNPs with grouped non-zero effects, and phenotype simulated considering a heritability *h*^2^ = 0, 5. Tournaments used groups of size *p*_*g*_ = 25.

It can be seen from Fig 4 that although tournament methods (C and D) have selected many zero effect markers with high frequencies, true (or close to) non-zero effect markers are also among the markers selected with high frequencies. The tournament methods (C and D) selected true non-zero effect markers with higher frequency than Bayesian Lasso (B), suggesting the superiority of tournament methods for marker selection. Tournaments with random groups (C) and tournaments with chromosome-conditioned groups (D) selected practically the same markers and at very similar frequencies, suggesting that the way the groups are formed in the tournament (randomly or chromosome-conditioned) has no influence on selection of markers. In addition, the high frequencies with which specific groups of markers are selected using tournaments suggests that the tournament method tends to always select the same markers in the end.

For the other scenarios considered in the simulation study, especially for the scenarios of dispersed non-zero effect markers, the difference between tournaments and Bayesian Lasso was even greater in relation to selection of markers, because the frequencies with which the tournament methods selected the true (or close to) non-zero effect markers were much higher than the frequencies with which Bayesian Lasso selected these markers. This fact directly influences GBV prediction, because the tournaments selected markers more efficiently than Bayesian Lasso. Thus, the GBVs predicted from the markers selected by tournaments will be much more similar to true GBVs (simulated) than the GBVs predicted from the markers selected by Bayesian Lasso.

### Influence of group size on GBV prediction

For the heritability *h*^2^ = 0, 25, the correlations between predicted GBVs and simulated GBVs were a little larger when the markers used in prediction of the GBVs were selected by tournaments with groups of size *p*_*g*_ = 25 in relation to groups of other sizes (50 and 100). As for higher heritabilities (0.5 and 1.0), group size in the tournaments (*p*_*g*_) practically did not influence correlations between predicted GBVs and simulated GBVs. Furthermore, the influence of groupsize depended on the number of SNPs selected at the end of the tournament, because when few SNPs were selected (near 100), the influence of group size was greater than when many SNPs (more than 4000) were selected.

The tournaments whose results are shown in the following sections used groups of size *p*_*g*_ = 25 since this group size exhibited better results in some of the situations considered in the simulation study.

### GBV predictive ability

**Fig 5:**
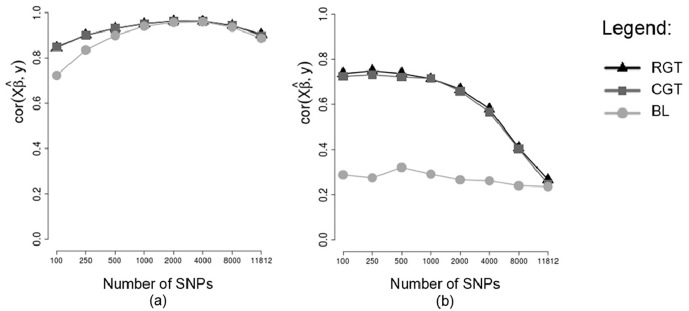
Means of the correlations between predicted GBVs and phenotypes. Analyzes without cross-validation (a) and in testing sets of an 8-fold cross-validation scheme (b), considering markers selected by different methods, considering a heritability of 0.25.

**Fig 6:**
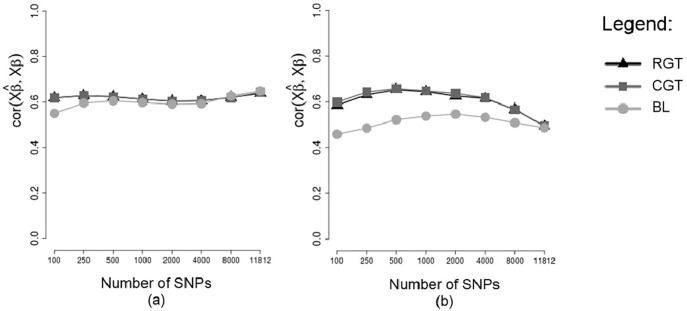
Means of the correlations between predicted GBVs and simulated GBVs. Analyzes without cross-validation (a) and in testing sets of an 8-fold cross-validation scheme (b), considering markers selected by different methods, considering a heritability of 0.25.

Correlations between predicted GBVs and phenotypes ($r_{G\hat{B}V,y}$) and correlations between predicted and simulated GBVs ($r_{G\hat{B}V,GBV}$) are shown respectively in Figs 5 and 6, obtained without using cross validation (a) and in testing sets of a cross validation scheme (b), considering different methodologies (RGT: Random Groups Tournaments, CGT: Conditioned Groups Tournaments and BL: Bayesian Lasso), in the scenario of 250 SNPs of non-zero effects, and considering a heritability of 0.25. Note that without cross-validation (Figs 5a and 6a) the increase in the number of markers caused a increase in correlations, this is due to the fact that individuals who had predicted GBVs were also used to fit the model, so errors in the data were probably erroneously explained by the markers. In the testing set of the cross validation scheme (Figs 5b and 6b) the increase in the number of markers caused a decrease in the correlations, this is due to the fact that individuals of the testing set were not used to fit the model. Thus, we can see that the use of thousands of markers in the model (without using a preliminary marker selection procedure) reduces the predictive ability in other individuals, which corroborates with [12]. Note also that without using cross-validation (Figs 5a and 6a) the Tournament methodology and Bayesian Lasso performed similarly, however, in the testing set of a cross-validation scheme (Figs 5b and 6b) the Tournament methodology outperformed Bayesian Lasso. One hypothesis that explains this is that the Tournament methodology selects markers more efficiently than Bayesian Lasso.

**Fig 7:**
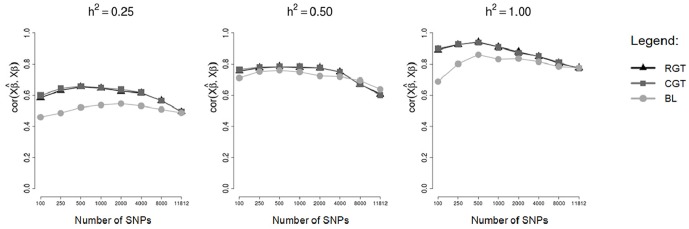
Mean correlations between predicted GBVs and simulated GBVs in testing sets. Means of the correlations in the testing sets of an 8-fold cross-validation scheme, considering markers selected by different methods and different heritabilities.

**Fig 8:**
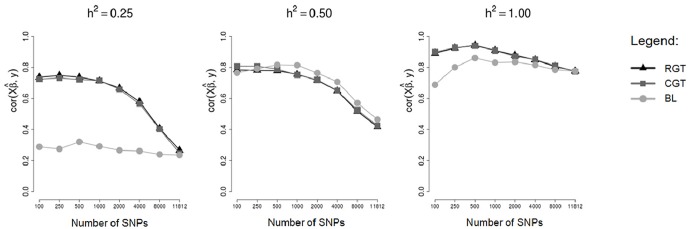
Mean correlations between predicted GBVs and phenotypes in testing sets. Means of the correlations in the testing sets of an 8-fold cross-validation scheme, considering markers selected by different methods and different heritabilities.

To compare the performance of the methodologies in different heritabilities the correlations between predicted and simulated GBVs ($r_{G\hat{B}V,GBV}$) and between predicted GBVs and phenotypes ($r_{G\hat{B}V,y}$) are show respectively in Figs 7 and 8, for testing sets of a cross-validation scheme, for markers simulated in a scenario of 250 SNPs with dispersed non-zero effects.

Note that for the heritability *h*^2^ = 0.25, there was a big difference between tournaments and Bayesian Lasso for practically all the numbers of markers used in fitting the model, however for the heritability *h*^2^ = 0.5 there was practically no difference between tournaments and Bayesian Lasso. Thus, we see that for low heritabilities, the markers selected by tournament methods were able to predict the GBVs in a more efficient way than the markers selected by Bayesian Lasso. Although there were not expressive differences between tournaments and Bayesian Lasso for the heritability *h*^2^ = 0.5, when we compare the methods in the absence of error (*h*^2^ = 1, 0), we observe that the tournament methods truly were superior to Bayesian Lasso.

As was already expected, both tournament methods (with random groups and chromosome-conditioned groups) practically did not differ from each other. Once more, this is due to the fact that the markers selected by both tournament methods were very similar, and so the GBVs predicted were also very similar.

For the scenario of 250 SNPs of non-zero effects, mainly for the heritability of 0.25, the correlations $r_{G\hat{B}V,GBV}$ (Fig 7) and $r_{G\hat{B}V,y}$ (Fig 8) calculated using GBVs predicted from SNPs selected by the tournament methods were much greater than the correlations calculated using GBVs predicted from SNPs selected by Bayesian Lasso. However, in the other scenarios (48 SNPs with clustered non-zero effects and 48 SNPs with dispersed non-zero effects) such a big difference was not observed between tournaments and Bayesian Lasso in regard to the same correlations. This is probably due to the fact that in the scenarios of 48 SNPs with clustered non-zero effects and 48 SNPs with dispersed non-zero effects, the non-zero SNPs were simulated with very large effects (Figs 1 and 2), which may have made the detection of these SNPs easier for all the methods.

### Analyses with real phenotypes

Using the real phenotypes of REA of the 384 animals and the genotypes of 530,566 SNPs, a marker selection stage was carried out with each one of the methods: random group tournaments, chromosome-conditioned group tournaments, and Bayesian Lasso. Groups of markers selected with different numbers of markers—100, 250, 500, 1,000, 2,500, 5,000, 10,000, 25,000, 50,000, 100,000, 250,000, and 530,566—were formed from classification of the markers generated by each selection method.

Using a cross-validation scheme, with eight groups of 48 animals, models were fitted to predict GBVs. These models were fitted to the sets of markers selected (of different numbers) by each method. Models for prediction of GBVs were fitted using Bayesian Lasso, whether the markers had been selected by Bayesian Lasso or by tournaments. In each step of cross validation, correlations were calculated between the predicted GBVs and the phenotypes of REA for the training and testing sets. Then the means of the correlations in the training sets and in the testing sets obtained in the eight steps of cross validation were calculated.

**Fig 9:**
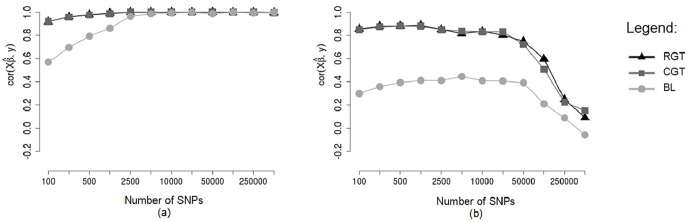
Mean correlations between predicted GBVs and real phenotypes of REA. Means of the correlations in training sets (a) and testing sets (b) of an 8-fold cross-validation scheme, considering markers selected by different methods.

The mean correlations between predicted GBVs and phenotypes for the training sets are shown in Fig 9a and testing sets in Fig 9b, considering different numbers of markers used for prediction of the GBVs and different marker selection methods.

The GBVs predicted based on markers selected by tournament methods exhibited correlations with the phenotypes (REA) much higher than the correlations between phenotypes and GBVs predicted based on markers selected by Bayesian Lasso, both for training sets (Fig 9a) and for testing sets (Fig 9b). Furthermore, in the testing sets, this difference was much more expressive. A very close similarity is seen between the correlations $r_{{G\hat{B}V}_{test},y_{test}}$ for the real data (Fig 9b) and the same correlations for the simulation study in the scenario of 250 SNPs with dispersed non-zero effects and heritability *h*^2^ = 0.25 (Fig 5b). In this situation of the simulation study, we saw that although the differences between tournaments and Bayesian Lasso were very large for the correlations between predicted GBVs and phenotypes, ($r_{{G\hat{B}V}_{test},y_{test}}$) (Fig 5b), these differences were not so large when it comes to correlations between predicted GBVs and true GBVs, ($r_{{G\hat{B}V}_{test},GBV_{test}}$) (Fig 6b). Since for real phenotypes we do not know the true GBVs of the animals, we cannot calculate the correlations between predicted GBVs and true GBVs ($r_{{G\hat{B}V}_{test},GBV_{test}}$). Nevertheless, taking into consideration the similarity between the analyses with real phenotypes and the analyses of the situation mentioned of the simulation study, we can suppose that for the real data of REA, the differences between tournaments and Bayesian Lasso also should not be so large when the correlations $r_{{G\hat{B}V}_{test},GBV_{test}}$ are considered.

Moreover, in Fig 9, we can observe that also in the analyses with real phenotypes, the tournament methods (with random groups and chromosome-conditioned groups) practically did not differ from each other, reinforcing even more the conclusions we had obtained in the simulation study.

**Fig 10:**
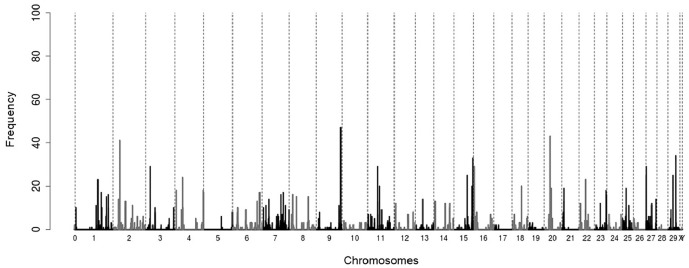
SNPs selected using an additional marker selection procedure. Frequencies with which the SNPs were selected in 100 analyses of real data of REA, using the tournament method and, subsequently, using HPDs of 95% for marker selection.

For the real data, after marker selection by tournaments, an additional marker selection procedure was applied. This procedure was based on fitting models, using Bayesian Lasso, to the markers selected in the tournament and determining HPD (Highest Posterior Density) credibility intervals of 95% for the estimates of the marker effects. That way, only the markers whose HPDs did not include zero were effectively selected. For the real phenotypes of REA, 100 replications of the step of marker selection by random group tournaments were made. Thus, in each one of these replications, the additional procedure of marker selection by HPDs of 95% was applied. The frequencies with which markers were selected by tournaments and HPDs in 100 analyses are shown in Fig 10. As the markers selected by HPDs differ significantly from zero at a level of 95%, it is thus probable that a marker selected by HPD and with high frequency in the 100 replications is truly a marker associated with the phenotype of REA or that it is in linkage disequilibrium with some marker associated with the phenotype.

Sets of markers were taken varying the minimum frequency with which they were selected in the 100 analyses, and for each one of these sets of markers, a model was fitted for prediction of GBVs used in a cross-validation scheme with eight groups of 48 animals.

Using only the 24 markers that were selected with higher frequencies we obtained correlations between predicted GBVs and phenotypes of REA of 0.7523 in training sets and 0.7058 in testing sets. Increasing the number of markers selected (more than 100 markers), the correlations draw near to 1.0 in the estimation group and exceed 0.9 in the validation group. Information on the 24 markers selected by tournaments with random groups and HPDs of 95% in at least 18 analyses are shown in Table 1.

**Table 1:** 24 SNPs selected with higher frequencies by random group tournaments and HPDs of 95%.

Name	Chromosome	Position	SNP
BovineHD4100000573	1	97662495	T/C
BovineHD0200008428	2	28831942	T/C
BovineHD0300005864	3	18193734	T/G
BovineHD0400001777	4	6215220	T/G
BovineHD0400009461	4	33371954	T/G
BovineHD0400034715	4	118478468	A/G
BovineHD0900029412	9	101146788	A/G
BovineHD1100011626	11	39328899	T/C
BovineHD1100013929	11	47541260	A/G
BovineHD1500016773	15	58042511	A/G
BovineHD1500022554	15	77321996	T/G
BovineHD1500024102	15	82626664	T/C
BovineHD1600001046	16	3676628	A/C
BovineHD1800010557	18	34671943	T/C
BovineHD2000007323	20	24343454	T/C
BovineHD2000008575	20	29153190	T/C
BovineHD2100002903	21	11682051	T/C
Hapmap31553-BTA-86073	22	26002227	A/G
BovineHD2300014081	23	48525753	A/G
BovineHD2500003540	25	12609037	T/C
BovineHD2700000410	27	1285441	T/C
BovineHD2700000415	27	1291103	T/C
BovineHD2900006510	29	22862702	A/G
BovineHD2900010094	29	33999692	T/C

For the real genotypes of REA, the times for execution of the marker selection step were:

- Random group tournaments: 11 minutes;
- Chromosome-conditioned group tournaments: 4 hours and 14 minutes;
- Bayesian Lasso: 3 hours and 8 minutes.

## Concluding remarks

The tournament method tends to consistently select the same markers in repeated analyses, whereas Bayesian Lasso tends to select different markers.

As the markers selected by tournaments were superior to those selected by Bayesian Lasso in relation to the predictive ability of GBVs, we conjecture that SNPs selected by tournaments have greater probability of being associated with the phenotypic trait.

In a context of GWAS, after reducing the number of SNPs using the tournament method, the model can be fitted using Bayesian Lasso, and then credibility intervals can be used to perform an additional procedure of marker selection. This procedure can provide greater evidence regarding the true SNPs associated with the trait under study.

The two modes of group formation in the tournament method evaluated in this study, random groups and chromosome-conditioned groups, practically did not differ from each other regarding the markers selected at the end of the tournament and, consequently, also did not differ in regard to the GBVs predicted from these SNPs.

As the tournament method using random groups was much more efficient than the other methods evaluated in relation to time for carrying out analysis, the random group tournament method can be recommended as the best among the methods evaluated in this study.

## Supporting information

S1 File Description and access to the phenotypic and genotypic data. This file contains description of the phenotypic and genotypic data and their accession information.
(PDF)

S2 File R script for tournament analysis. This file contains a custom R script to perform Tournament Analyses (to reduce the set of SNPs to a desired level).
(R) Click here for additional data file.

S3 File R script for GBVs prediction into a cross validation scheme. This file contains a custom R script to use the SNPs selected by Tournaments to fit predictive models using Bayesian Lasso, into a cross validation scheme.
(R) Click here for additional data file.
