# Correction: Tournaments between markers as a strategy to enhance genomic predictions

**DOI:** 10.1371/journal.pone.0219448

**Published:** 2019-07-05

**Authors:** Diógenes Ferreira Filho, Júlio Sílvio de Sousa Bueno Filho, Luciana Correia de Almeida Regitano, Maurício Mello de Alencar, Rosiana Rodrigues Alves, Marielle Moura Baena, Sarah Laguna Conceição Meirelles

Marielle Moura Baena is not included in the author byline. Marielle Moura Baena should be listed as the sixth author and affiliated with Departamento de Zootecnia, Universidade Federal de Lavras, Lavras, Minas Gerais, Brazil. The contributions of this author are as follows: Data Curation, Formal Analysis, Investigation.
