# Supplementary material for: Tournaments between markers as a strategy to enhance genomic predictions
Source: PLoS One. 2019 Jun 24;14(6):e0217283. doi: 10.1371/journal.pone.0217283 (PMC6590785; doi:10.1371/journal.pone.0217283)
Supplement: S1 File — This file contains description of the phenotypic and genotypic data and their accession information. (PDF) [file pone.0217283.s001.pdf]

S1 File - Description and access to the phenotypic and genotypic data

This file contains description of the phenotypic and genotypic data used in the paper "Tournaments between markers as a strategy to enhance genomic predictions" published in Plos One, and the accession information.

phenotypes.RData

This RData object contains the vector of phenotypes after quality control and corrected for the contemporaries groups effect (described in the "Material and methods" section).

genotypes.RData

This RData object contains the genotype matrix after the quality control (described in the "Material and methods" section). Because the main purpose of the Tournament methodology is to reduce the set of markers to improve genetic predictions (ie, this is a prediction study) and not to promote a final marker selection method (other additional methodologies are needed to make a final selection of markers) there is no information on SNPs positons.

Data availability

The files phenotypes.RData and genotypes.RData can be accessed in `https://figshare.com` using DOI: 10.6084/m9.figshare.8171351. These files can also be accessed directly from the link: `https://figshare.com/s/c896d5cfe86ca8b7328a`.
